# Supplementary material for: Characterization of Spontaneous Melanization by Fluorescence Spectroscopy: A Basis for Analytical Application to Biological Substrates
Source: Biology (Basel). 2023 Mar 11;12(3):433. doi: 10.3390/biology12030433 (PMC10044826; doi:10.3390/biology12030433)
Supplement: Supplementary file 1 [file biology-12-00433-s001.zip › biology-2267077-supplementary.pdf]

Supplementary information

Characterization of spontaneous melanization by fluorescence spectroscopy: a basis for analytical application to biological substrates

Anna Cleta Croce[1*] and Francesca Scolari[1,]

[1] Institute of Molecular Genetics, Italian National Research Council (CNR), Via Abbiategrasso 207, and Department of Biology & Biotechnology, University of Pavia, Via Ferrata 9, I-27100 Pavia,
* Correspondence: croce@igm.cnr.it.it; Tel.: +39-0382-986-428

Content | Page

Content	Page
Supplementary details on the spectral fitting analysis procedure	1
Figure S1	2
Figure S2A,B	3
Figure S3A,B	4

Supplementary details on the spectral fitting analysis procedure

The emission bands detected from the biological sample solutions under excitation at 220 nm were analyzed by means of a curve-fitting procedure, based on the Marquardt-Levenberg algorithm (PeakFit; SPSS Science, Chicago, IL). The procedure was first applied to analyze the emission bands of L-tyrosine and L-tryptophan, to define the parameters describing at the best their profile, in terms of respective Half-Gaussian Modified Gaussian (GMG) functions. The GMG functions so defined were then applied to analyze the emission bands obtained from the biological samples under excitation at 220 nm. The procedure was based on finding the minimum value of the sum of squared deviations. The processing required two additional spectral components, one peaking at about 315 nm, and one peaking at about 410-420 nm, for a complete achievement of the goodness of fitting. Subsequent adjustments based on the linear combination of the spectral function were performed, until the best fit between the sum of their respective contributions and the real spectral profile was achieved. Before being submitted to the fitting procedure, wavelengths were converted in wave-numbers, because the theoretical models for line spreading are based on frequency. At the end of fitting, measured spectra and the GMG defined curves were reconverted to wavelengths for a more familiar presentation. Before being submitted to the fitting procedure, the peak maximum values of the spectra were also normalized to the value of 100 a.u., and the relative contribution of each component to the overall emission was provided as the integrated area of the respective band. The goodness of the fitting of each spectrum was verified from the analysis of the residuals and the assessment of the coefficient of determination (r^2).

FIGURE S1

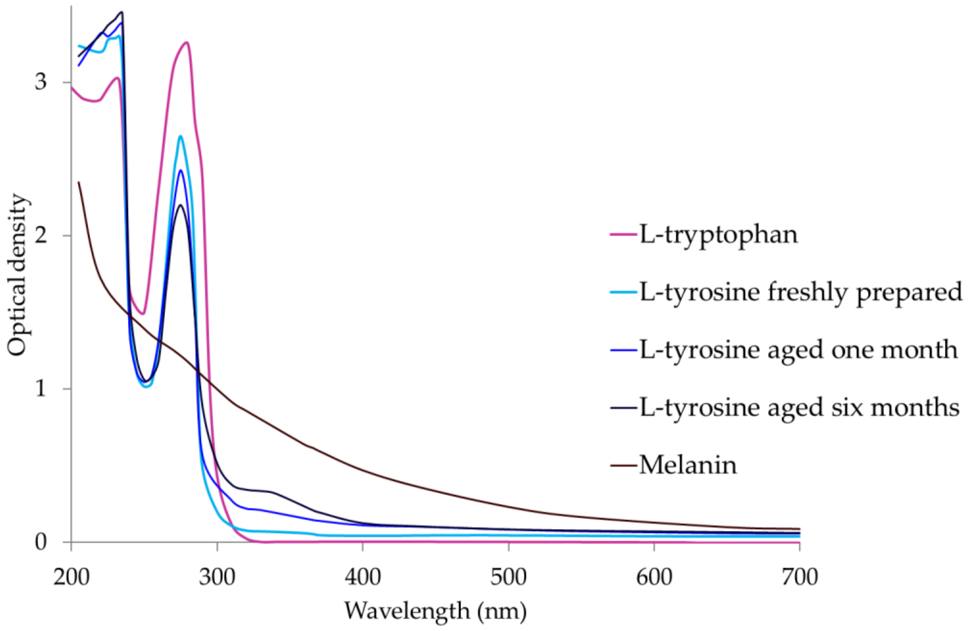

Figure S1 – Absorption spectra of melanin, L-tyrosine (1.5 mM, phosphate buffer solution -pH 7.2, 0.1 M; immediately after preparation and at different times of ageing), and of L-tryptophan (0.6 mM, phosphate buffer solution -pH 7.2, 0.1 M) as indicated by the colors, on the right.

FIGURES S2,A,B

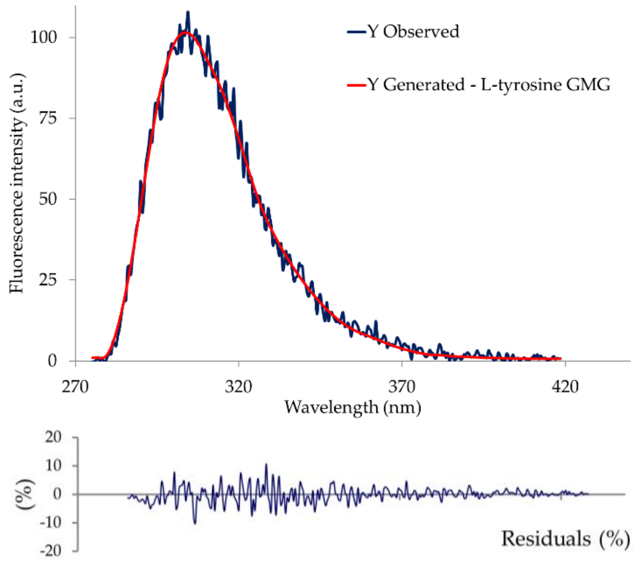

Figure S2,A – Assessment of the GMG function representing L-tyrosine fluorescence emission spectrum under excitation at 220 nm. Goodness of fitting verified by analysis of residuals (shown below the spectrum), and coefficient of determination ($r^2 \geq 0.994$).

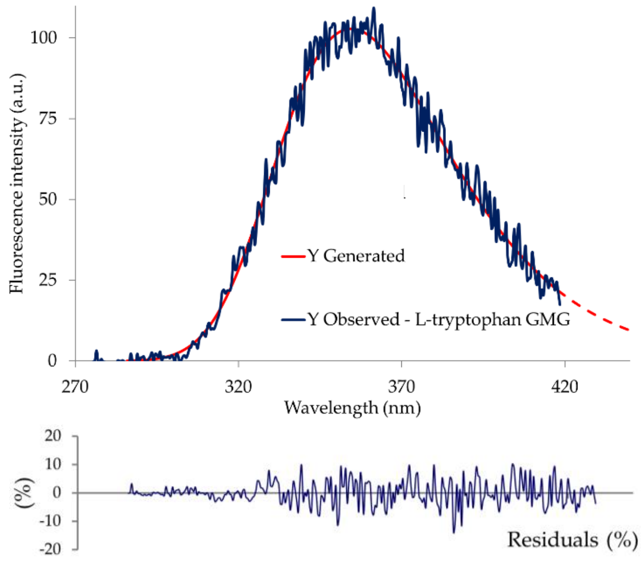

Figure S2, B – Assessment of the GMG function representing L-tryptophan fluorescence emission spectrum under excitation at 220 nm. Goodness of fitting verified by analysis of residuals (shown below each respective spectrum), and coefficient of determination ($r^2 \geq 0.983$).

FIGURES S3,A,B

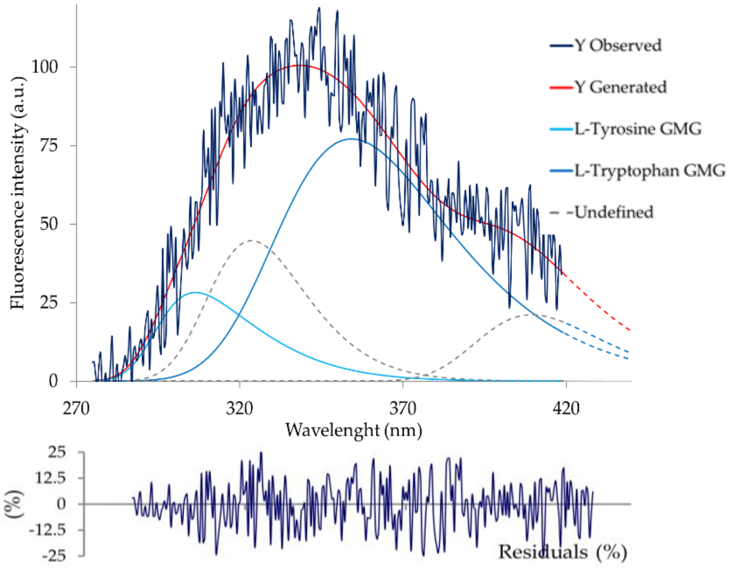

Figure S3, A – Fitting analysis of the fluorescence emission spectrum recorded from black cat hair aqueous extract under excitation at 220 nm. Goodness of fitting verified by analysis of residuals (shown below each respective spectrum), and coefficient of determination ($r^2 \geq 0.901$).

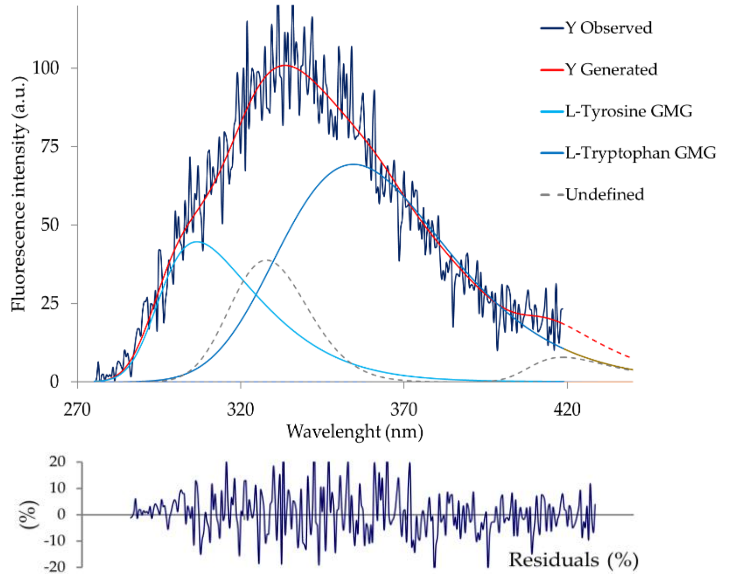

Figure S3, B – Fitting analysis of the fluorescence emission spectrum recorded from mosquito egg aqueous extract under excitation at 220 nm. Goodness of fitting verified by analysis of residuals (shown below each respective spectrum), and coefficient of determination ($r^2 \geq 0.943$).
